# Quality monitoring in the English National Health Service

**DOI:** 10.1186/1750-1172-9-S1-O3

**Published:** 2014-11-11

**Authors:** Edmund Jessop

**Affiliations:** 1 Specialised services team, NHS England, London SE1 6LH, UK

## 

Monitoring of services in the National Health Service in England is framed around clinical outcomes, safety and patient experience.

A system of dashboards is being developed to provide monthly information about specialised services. The initial pilot has included a set of key information about services to patients with haemophilia and cystic fibrosis, for example the proportion of patients on home treatment (haemophilia) or the proportion admitted to a ward with specialist staff (cystic fibrosis). Data items were agreed by clinicians as relevant and important. The information is available from every provider.

In the highly specialised services for very rare disease (prevalence less than 1 in 100 000) the dashboard runs annually, not monthly, and is based on long experience [1]. Outcome monitoring is particularly well developed for sold organ transplant, with agreed protocols for investigating and responding to statistical outliers.

Quality of life measures are used in some services such as epidermolysis bullosa and neurofibromatosis type 2 [2], but there is not enough experience with these measures to compare different hospitals or to track trends.

Safety incidents such as ‘never’ events are rare; they are reported and investigated immediately.

Patient experience is monitored through surveys and close contact with patient organisations.

A key mechanism for monitoring quality in the highly specialised services is an annual meeting. Attendance by all providers of the service is mandatory. Clinical outcome data is presented on all patients treated, and interesting or informative cases are discussed by the clinical teams. The meeting provides an excellent forum for peer review, exchange of clinical experience and learning.
